# Bridging perspectives: Success factors for AI implementation in healthcare from healthcare professionals and AI experts

**DOI:** 10.1177/20552076261437277

**Published:** 2026-03-25

**Authors:** Zohreh Yousefi Dahka, Timo Koivumäki

**Affiliations:** 1 6370 University of Oulu, Oulu Business School, Martti Ahtisaari Institute, Oulu, Finland

**Keywords:** AI in healthcare, AI implementation, key success factors, AI experts, healthcare professionals, NASSS framework

## Abstract

**Objective:**

The integration of Artificial Intelligence (AI) in healthcare offers opportunities to transform patient care, improve efficiency, and support clinical decision-making. Yet, its implementation is hindered by technical, organizational, and collaborative challenges. This study explores the key success factors for sustainable AI adoption from the perspectives of healthcare professionals (HCPs) and AI experts, with the aim of identifying alignments and gaps that influence collaboration and sustainability.

**Methods:**

Guided by the NASSS framework, semi-structured interviews were conducted with four HCPs and five AI experts with experience related to AI technologies in the healthcare sector. Thematic analysis was applied to examine stakeholder perspectives, focusing on gaps and alignments between stakeholders and identifying common patterns.

**Results:**

Transparency leading to trust was the most emphasized factor by both AI experts and HCPs. Alignments were also found in recognizing the importance of interorganizational cooperation, demand-side value, scope of use, usability, responsibility and redefining the roles. Gaps included challenges in cooperation with HCPs, misunderstandings between stakeholders and the need for interdisciplinary experts, insufficient training, concerns over data quality and privacy, and limited attention to usability. Based on the found patterns, a framework is proposed to strengthen collaboration between AI experts and HCPs, enabling effective and sustainable AI implementation in healthcare.

**Conclusion:**

This study extends the NASSS framework to a dual-stakeholder context, offering insights into the alignments and gaps between HCPs and AI experts. The proposed framework supports improved collaboration, guiding healthcare professionals, AI developers, and managers toward effective, sustainable AI implementation and fostering mutual understanding for successful adoption.

## Introduction

Health systems globally are facing challenges such as workforce shortages in patient care, rising costs, inadequate infrastructure, limited access to services, aging populations, and the emergence of new disease strains.^1–4^ The COVID-19 pandemic further highlighted these weaknesses, revealing shortages in resources, poor information sharing, deficiencies in diagnostic testing, and excessive workloads for frontline healthcare workers. Consequently, the growing demand for high-quality care must be balanced against the ongoing shortage of personnel and resources in the healthcare sector.^2^ In light of these challenges, artificial intelligence (AI) offers significant potential for the healthcare industry, particularly as a promising solution to mitigate the shortage of healthcare professionals while accommodating the growing patient population through innovative approaches.^2,4^ Gilson et al.^5^ found that ChatGPT, utilizing natural language processing, is capable of answering medical questions with a proficiency comparable to that of a third-year medical student in the United States.

AI has introduced a range of transformative applications aimed at improving healthcare services by offering valuable and supportive assistance, and its use in the healthcare sector is expected to grow due to the increasing complexity and volume of data in this field.^4,6,7^ Research shows strong demand within the healthcare sector with 86% of healthcare providers, as well as technology and life sciences companies using AI. AI applications are increasingly used in clinical trials (22.7%), while radiology accounts for the largest share in therapeutic area support (75%).^8^

However, fully realizing the benefits of AI in healthcare requires effective implementation, which entails addressing multiple major challenges. This process involves not only retraining the workforce and reorganizing health services but also navigating various legal, ethical, and social issues.^9^ AI and digital technologies have been found to exert a moderate to substantial influence on 13 health-related sub-goals and six other UN Sustainable Development Goals (SDGs). This demonstrates their pivotal role in advancing the SDGs, particularly the third goal in healthcare, which aims to ensure universal health coverage and improve global well-being, with a special focus on developing countries.^10–12^ Implementing AI tools and ensuring their sustainable use is a complex process, as such technologies often encounter skepticism stemming from concerns over algorithmic and data bias, limited clinical involvement in product development, safety and risk issues, liability, legal and ethical considerations, and potential effects on professional roles.^13–15^

Therefore, the implementation of AI in healthcare demands thorough planning and structured organization to guarantee quality, safety, and acceptance.^16^ In addition, interdisciplinary collaboration among stakeholders, such as medical practitioners, academic instructors, computer scientists, health informatics professionals, and other relevant experts, should be promoted throughout the development and deployment of AI technologies.^17^ Moreover, multiple studies have underscored the essential role of AI specialists and medical professionals in implementation efforts, stressing the importance of their collaborative engagement.^1,13,18^

Guided by the literature highlighting the significance of stakeholder collaboration and the necessity for supportive frameworks and regulations, this study addresses the scarcity of research on cooperation between healthcare professionals (HCPs) and AI experts. Although these groups work closely together, their differing viewpoints, especially regarding collaboration in the implementation of AI technologies in the healthcare sector, remain poorly understood, highlighting a significant research gap.

This study, a developed version of previous research,^19^ addresses this gap and seeks to examine how success factors align between healthcare professionals and AI experts in implementing AI within healthcare, using a qualitative research approach. The aim is to deliver practical insights into the healthcare sector by identifying success factors that should be preserved and pinpointing gaps that require attention. Additionally, the research intends to support the creation of a framework for managing interactions between HCPs and AI experts, with particular emphasis on Finland. The Nordic countries are widely regarded as leaders in digital health technologies, with national implementation guided for many years by sector-specific strategies, and Finland stands out as particularly ambitious in its aim to transform its health system through digitalization.^20^ The study addresses one main question, divided into two sub-questions:

• What are the key success factors from the viewpoints of healthcare professionals and AI experts?
• What gaps exist between HCPs and AI experts concerning these success factors?

Answering these research questions has both theoretical and practical implications. Theoretically, the study extends the use of the NASSS framework by applying it to investigate collaboration between stakeholders during the implementation process, rather than examining its domains separately or analyzing stakeholders only within specific domains. Practically, addressing these questions clearly highlights the gaps between stakeholder groups that decision makers and policy makers need to focus on more during the implementation of AI technologies in the healthcare sector, as well as the areas of alignment that should be emphasized to ensure smooth implementation.

## Literature

### Challenges and success factors of AI implementation in healthcare

The growing number of digital health applications, rising expectations for progress in medical, social, and economic fields, and the adoption of digital health practices accelerated by COVID-19 have created a need to identify the key elements for successful AI deployment.^21^ However, although technologies are commonly viewed as a means to improve care by increasing safety and efficiency, in reality, such projects often fail to achieve the complete set of expected benefits.^22^

According to the literature, the gap between the potential and actual impact of AI technologies in medical settings may be attributed to the historical focus on technology development, market entry, and commercialization. The divide between research and real-world application largely arises from the challenges of implementing AI solutions, which demand careful consideration of the specific conditions and factors necessary for effective integration into routine clinical practice.^23,24^

The challenges surrounding AI implementation are global in nature, which have prevented AI from achieving widespread adoption in healthcare practice.^24^ AI implementation in healthcare can be described as a multifaceted socio-technical initiative, as its success in clinical settings depends on more than technical performance and involves diverse stakeholders, including technology creators, regulatory agencies, healthcare organizations, professionals, patients, and care providers. This level of intricacy places AI toward the more challenging end of the spectrum compared to traditional measures explored in implementation science.^25,26^

Several studies have examined the challenges and success factors linked to implementing AI technologies in the healthcare sector. One key challenge involves data-related issues, such as gaining access to large volumes of data, inconsistencies in healthcare data, and concerns regarding data privacy and confidentiality.^24,27,28^ Another challenge highlighted in several studies concerns legal issues and regulatory frameworks, including compliance with established guidelines, gaps in existing policy structures, regulatory questions about accountability for errors in AI-assisted decision-making, and technology-related difficulties.^21,24,27–29^ Furthermore, the literature highlights concerns related to stakeholders in the implementation process, including the need for active engagement of all parties involved and the varying attitudes toward adopting AI in healthcare.^24,25,27^ Past research has also identified technology and assessment-related challenges, including the need for seamless integration into complex clinical workflows, issues with health system infrastructure, the lack of sufficient medical and economic impact evaluations, and differing opinions on how to assess the value of AI.^21,24,27,29^ Some studies have also pointed out research-related challenges in this field, such as the scarcity of real-world evidence and inadequate funding mechanisms, along with the need for further studies to produce insights essential for developing implementation frameworks that can support the future deployment of AI in clinical settings.^25,29^

**Figure 1.**
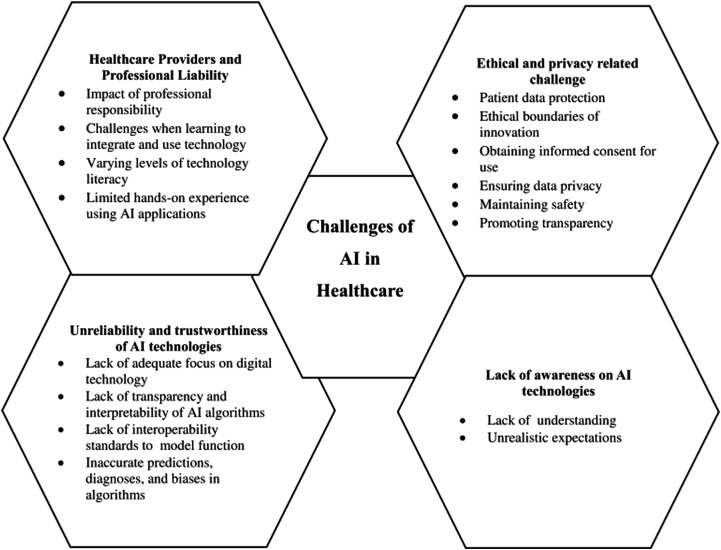
Challenges of healthcare AI implementation reported by Wubineh et al.^1^

In their comprehensive literature review, Wubineh et al.^30^ categorize the challenges of AI implementation in healthcare into four main themes: (1) moral and confidentiality concerns, primarily related to data usage and informed consent; (2) lack of awareness about AI applications, referring to insufficient knowledge that can lead to unrealistic expectations; (3) concerns about the unreliability and credibility of AI technologies, focusing on the lack of transparency in algorithms and inaccurate predictions; and (4) healthcare professionals and responsibility, highlighting the need for training and clarifying the responsibilities of healthcare professionals in using AI and interpreting its results. The specific barriers under each theme are illustrated in Figure 1.

### NASSS framework and AI technologies in healthcare

The Nonadoption, Abandonment, Scale-up, Spread, and Sustainability (NASSS) framework was suggested by Greenhalgh et al.^31^ in the study to develop a practical, evidence-based framework that can help predict and assess the success of technology-supported health or social care programs. The resulting NASSS framework, developed through literature review and case studies, covers seven key domains. These include the Condition, which covers clinical, comorbidity, and sociocultural factors affecting patient suitability; Technology, which examines ease of use, reliability, data impact, and sustainability risks; Value Proposition, which weighs business viability against patient benefits; Adopter System, which focuses on acceptance by staff, patients, and caregivers and the factors influencing their support; Organization(s), which looks at readiness, resources, and coordination for adoption; Wider Context, which considers policy, regulatory, and financial influences; and Embedding and Adaptation Over Time, which stresses the importance of continuous evaluation and flexibility for long-term success.^31^

**Figure 2.**
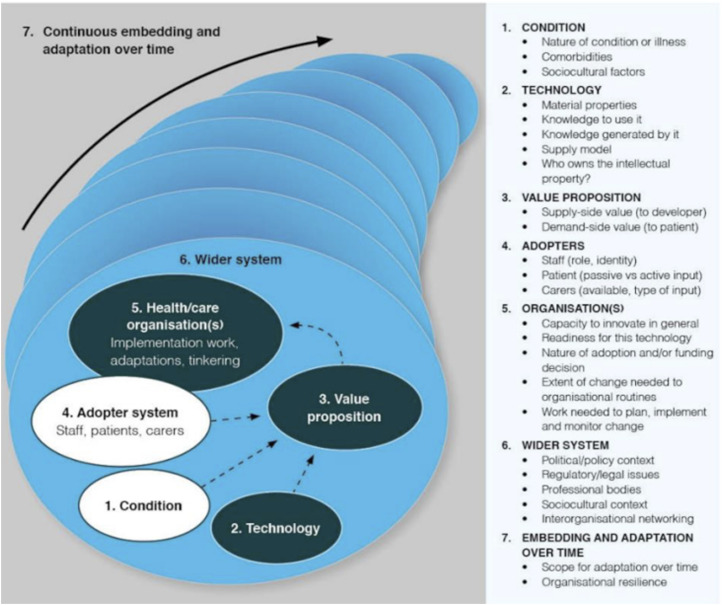
The NASSS framework, developed by Greenhalgh and Abimbola (2019), adapted from Greenhalgh et al. (2017).

Greenhalgh and Abimbola^22^ expanded the NASSS framework from its 2017 version by adding new subdomains. The Condition domain gained a “Sociocultural factors” subdomain to capture cultural and societal influences on use. The Technology domain added “Who owns the intellectual property”, emphasizing issues of data and technology ownership. In the Wider System domain, “Interorganizational networking” was introduced to address the collaborative relationships between organizations involved in implementation. The updated version of the framework is shown in Figure 2.

The NASSS framework was systematically developed to address gaps in the literature concerning technology implementation. It focuses not only on adoption but also on nonadoption and abandonment, as well as the challenges in transitioning from a local pilot project to full-scale integration (scale-up), expanding to other settings (spread) and ensuring long-term sustainability through ongoing adaptation to the changing context.

While the NASSS framework was not originally developed for AI, it effectively guides implementation in healthcare. Several studies have successfully applied it to AI integration in healthcare. Reddy^32^ showed that assessing the NASSS elements reveals key barriers and facilitators to adopting generative AI and provides an effective lens for analyzing complex adaptive systems. Hogg et al.^18^ also identified NASSS as the most suitable tool for synthesizing evidence in their systematic review of AI implementation. Nair et al.^33^ reported that factors influencing adoption of an AI-based clinical decision support system align well with its seven domains. Strohm et al.^34^ also used NASSS in a qualitative study on clinical radiology, comparing interview data with the framework and creating a modified version that retained main domains while tailoring subdomains to the study context. Importantly, NASSS also incorporates sustainability in implementation—an increasingly critical issue as AI adoption grows—aiming to maximize benefits while supporting environmental preservation.^10,31,35^

Regarding the implementation of AI technologies in healthcare, with a focus on stakeholder collaboration, the NASSS framework has priority over other frameworks. Compared to TAM^36^ and its extension UTAUT,^37^ NASSS considers different stakeholders in the domains of *Adopters* and *Wider system*, includes all steps of technology integration from design to use, is specifically developed for healthcare, and accounts for sustainability. In contrast, TAM and UTAUT focus mainly on individual-level determinants such as perceived usefulness, ease of use, and behavioral intention, while other implementation steps are overlooked.

The Practical, Robust Implementation and Sustainability Model (PRISM), developed by,^38^ incorporates the RE-AIM evaluation framework.^39^ PRISM integrates elements of program design, predictors of implementation and diffusion, and outcome measures, with emphasis on sustainability and stakeholder engagement. However, unlike NASSS, it is not tailored to healthcare technologies and instead covers interventions broadly, lacking technology-specific elements. The Consolidated Framework for Implementation Research (CFIR), developed by Damschroder et al.,^40^ also identifies factors influencing implementation in healthcare. Yet, while it includes stakeholder perspectives, it is not dedicated to technology in healthcare and, like PRISM, omits technology-specific considerations.

## Methodology

### Theoretical framework

The theoretical framework of this research is grounded in the NASSS framework, with a focus on selecting the domains, subdomains, and themes most relevant to HCPs, AI experts, and their collaboration. This selection is primarily guided by the application of the NASSS framework in Hogg et al.^18^ and their findings on factors considered important by both HCPs and AI experts, or by either group individually, complemented by insights from other relevant literature.

The study by Hogg et al.^18^ is selected as the basis for this research because it offers one of the most comprehensive analyses of clinical AI implementation in recent years. Through a systematic review of 111 qualitative studies (2014–2021), it consolidated the perspectives of key stakeholders, including HCPs, patients, developers, healthcare leaders, and policymakers. The study aimed to identify factors influencing AI adoption in healthcare and to highlight evidence gaps, using the NASSS framework as its foundation. Importantly, it extended the framework by introducing two subdomains: Care pathway positioning (Technology domain), which considers tool independence, interaction timing, and response context; and Relationships (Adopters domain), which examines how AI affects trust, communication, and collaboration among HCPs and their relationship with patients.

The theoretical framework of the study is in Table 1. The detailed selection criteria of each domain and subdomain are in appendix 1 of the paper.

**Table 1.** Theoretical framework for identifying key success factors in AI implementation in healthcare.

NASSS domain	NASSS subdomain	Themes
Condition	Nature of condition or illness	*No special theme*
	Comorbidities	*No special theme*
	Socio-cultural factors	*No special theme*
Technology	Material properties	*Usability of the tool*
	Knowledge to use it	*Agreeing the scope of*
	Knowledge generated by it	*Communicate meaning effectively*
	Supply model	*Quality of the health data and guidelines used*
	Care pathway positioning	*Extent of tools’ independence*
Value proposition	Demand-side value	*No special theme*
Adopters	Staff (role and identity)	*Appetite and needs differ between staff groups*
		*Tools redefine staff roles*
		*Aligning with staff values*
Organization	Extent of change needed to organizational routines	*Fitting the tool within current practices*
Wider Context	Professional bodies	*Lack of understanding between professional groups*
	Regulatory and legal issues	*Deciding who is responsible*
Embedding and Adaptation over Time	Scope for adaptation over time	*No special theme*
	Organizational resilience	*No special theme*

### Data collection

This study follows a qualitative research methodology which is consistent with previous studies that employed semi-structured interviews with stakeholders to identify key success factors for AI implementation in healthcare.^27–29,33,41^ The semi-structured interviews in this study were conducted with HCPs and AI experts from October to December 2024 in Oulu, Finland.

The questions were derived from the domains and subdomains of the NASSS framework, selected based on the literature (Appendix 1). They were designed to be general and exploratory to avoid biasing interviewees’ responses regarding each subdomain. The questionnaire was not based on a previously validated instrument; however, its relevance for the two studied groups was reviewed by an AI expert with experience in healthcare AI implementation to ensure clarity and relevance to interviewees’ roles and alignment with the intended subdomains and themes.

Purposive sampling was used to select participants with relevant expertise, as it targets respondents most likely to provide valuable insights.^42^ For this study, the inclusion criteria for healthcare professionals were doctors with experience in using or implementing AI in healthcare, while AI experts included developers or their supervisors involved in creating and applying AI tools in this field.

The interviews were conducted separately, with participants answering approximately 13–15 questions tailored to their expertise and guided by the study’s theoretical framework. In addition, an open-ended question was included to capture other potentially relevant factors, allowing for the identification of overlooked subdomains or the emergence of new subdomains and themes.

In total, nine interviews were conducted with participants based in Finland including four HCPs and five AI experts.^43^ Given the demanding schedules of many interviewees, particularly HCPs, both online and email formats were used. One HCP and three AI experts took part in live online interviews lasting 30 minutes to one hour, while the others completed email interviews. There were no relationships between the interviewer and the participants before the interview; however, the interview invitations sent by email included information about the interviewer’s current education and role, the research goals, and the importance of the study.

For online interviews, questions were sent in advance, and for email interviews, participants were asked to return their responses within one week. This approach allowed all interviewees, regardless of format, to reflect on the topics and provide more considered and comprehensive responses. Additionally, no follow-up questions were asked based on interviewees’ answers in online interviews, so the depth of responses stayed the same across both formats.

Informed consent was obtained verbally at the beginning of the interview, during which the interviewer read the privacy notice; if the interviewee agreed, the interview proceeded. In email interviews, the privacy notice was provided at the beginning of the interview, and the interviewee was asked to continue if they agreed to the terms. This procedure followed the publicly available guidelines of the University of Oulu Human Sciences Ethics Committee, in line with national TENK guidelines,^44^ which emphasize informed and voluntary consent rather than a specific format and allow verbal or electronic consent.

For both online and email interviews, participants were informed in advance about the ethical considerations, including the use and analysis of data, procedures for anonymization, and data retention. Informed consent was obtained before each interview. The online interviews were audio-recorded with participants’ consent and later transcribed using Microsoft Teams’ automated tool. Field notes were taken during and shortly after each interview. These notes included the interviewer’s reflections on emerging themes and were reviewed during data analysis to assess their relevance and consistency with the interview data. All transcripts were carefully checked against the audio recordings to correct any transcription errors and ensure accuracy. Details of the participants and interviews are in appendix 2 of the paper.

Demographic data such as age, gender, and education were not collected. In line with the General Data Protection Regulation (GDPR), personal data must be “adequate, relevant, and limited to what is necessary in relation to the purposes for which they are processed” (Article 5(1)(c)).^45^ As these demographic data were not required to address the study goals and were not included in the theoretical framework, they were excluded to protect participant anonymity and privacy. In addition, given the small sample size, collecting demographic data would not provide meaningful analytical insights. Instead, participants’ current professional roles and relevant experience were collected as background information to support the interpretation of perspectives.

### Data analysis

Data collection and primary coding and analysis were conducted by one author (ZYD), a doctoral researcher in Business Analytics with an interdisciplinary background in electrical engineering, business administration, and analytics, which may have shaped the analytical focus on technological and organizational aspects of AI implementation. To enhance analytical rigor and reduce potential bias, the analysis was reviewed and discussed with the supervising author (TK), an associate professor specializing in business analytics and digital service business with strong background in digital health, at the University of Oulu, who provided independent oversight and critical feedback throughout the research process.

This study employed thematic analysis to examine the qualitative interview data. Recommended by Braun and Clarke^46^ as a foundational qualitative method, it helps develop essential analytical skills. Thematic analysis seeks to identify meaningful patterns or themes that address research questions and provide deeper insights, going beyond description to interpret and explain the data.^47^

This study applies an abductive approach to thematic analysis. Abductive methodologies, as noted by Nenonen et al.,^48^ enhance research quality by producing concepts that are practical, understandable, and applicable to real-world contexts. Thompson^49^ further provided a structured guide for abductive thematic analysis, positioning it between inductive and deductive approaches by combining empirical data with theoretical frameworks. In this research, the NASSS framework was used to guide predefined subdomains and themes, while also allowing for the identification of new factors that could extend the framework. Following Thompson,^49^ this approach uses existing theory as a lens while staying open to novel insights, thereby avoiding arbitrary conclusions and exposing gaps where current frameworks may not fully explain empirical findings.

The qualitative data in this study were analyzed using thematic analysis with the aid of MAXQDA software. The choice of this software was informed by Weinert et al.,^41^ who also employed it to explore stakeholder perspectives on AI implementation in hospitals. The analysis was conducted by one analyst and subsequently checked and validated by another. To identify both gaps and alignments between the two stakeholder groups, HCPs and AI experts, the analysis was first conducted separately for each group and later combined to propose a framework aimed at improving collaboration between them.

Each interview transcript was initially read in full to gain an overall understanding of the participants’ views. Transcripts were then coded in MAXQDA using predefined codes derived from the NASSS framework, while additional inductive codes emerging from the data were incorporated into the coding scheme for use in subsequent transcripts. Once coding was completed, earlier transcripts were revisited to determine whether the newly added inductive codes applied. Inductive codes generated during the process were either mapped to existing NASSS subdomains where applicable or incorporated into the framework as new themes. The final analysis for each stakeholder group was structured around the NASSS framework’s subdomains and the themes identified in this study.

In addition, memos were created for each transcript to capture key insights and participant reflections, which supported the identification of patterns in the data. These emerging patterns were then examined across all participants and integrated into a final list, which led to the design of a framework in this study.

## Results

The following section presents the data analysis structured around the domains and subdomains of the NASSS framework.

### Domain: Condition

This domain was not strongly emphasized by AI experts in relation to their collaboration with HCPs. However, two participants noted that AI tools in healthcare require further improvement to address comorbidities, illness conditions, and socio-cultural factors. As one expert explained, “*…for example, here is some model that predicts something based on the head X-ray images or CT scans, but it’s all limited only for this age group and for this kind of pleadings and nothing else…*” (AI expert, Interview 4).

Two HCPs mentioned challenges with AI tools regarding the subdomain of “Nature of condition or illness”, and one raised concern about “comorbidities”. “*…the tool is very efficient but sometimes struggles with challenging cases*” (HCP, Interview 6). “*Whenever an image has multiple or subtle abnormalities or the image is dissimilar to those it has been trained with its performance drops to an unacceptable level…*” (HCP, Interview 9).

### Domain: Technology

#### Subdomain: Care pathway positioning

Regarding the theme “extent of tools’ independence”, most AI experts agreed that final patient-care decisions should rest with HCPs, as required by regulations. They noted that HCPs generally prefer retaining decision-making authority, with AI mainly providing support. However, one participant observed that in certain time-consuming tasks, some HCPs may prefer AI to handle the entire process: “*But like a good example is to measure a tumor in a medical image so that’s hard, and it takes time, so the radiologists don’t want to do that…they kind of want the tool to be fully independent there completely*” (AI expert, Interview 5).

HCPs expressed mixed views on full automatization. Two participants felt it could be acceptable in certain circumstances, while two others emphasized that final decisions should remain with clinicians. As one noted, “*You can doubt it...we need to see the conclusion and that’s why AI never makes the decision itself. If we cannot follow the logic, we cannot let it make the decision*” (HCP, Interview 7). Another added, “*Complete automatization is fine if feedback is given to develop the AI application*” (HCP, Interview 6).

#### Subdomain: Supply model

For the theme “quality of health data”, AI experts generally did not view data issues as a major challenge in their collaboration with healthcare providers. However, one participant noted that HCPs expressed concerns about the location of stored data, “…*Someone has said that you need to be sure that the data is located within the EUE TA area and region…”* (AI expert, Interview 1).

Two HCPs commented on this factor. One noted that trust in AI experts to access the required data operates more at the organizational level, while another raised concerns about security and privacy. Both also highlighted challenges with training data. As one explained, “…*There is also the concern of patient information security, and I presume all data that is assessed by AI is also used to train it*…” (HCP, Interview 9). Another stressed the importance of local validation: “*It is important to test that models really work and with local data*” (HCP, Interview 8). A further comment emphasized performance gaps, *“*…*the real-life performance of AI tools is never what is being marketed, as the real-life dataset is different from the training dataset*” (HCP, Interview 9).

#### Subdomain: Knowledge generated by it

Regarding the theme “communicate meaning effectively”, several AI experts stressed that HCPs need transparency in how AI systems make decisions, especially in patient care. Some linked this to the “black box” problem, which they felt fosters distrust, while one expert argued that demonstrating clinical evidence of effectiveness is sufficient reassurance. As one participant explained, “*The healthcare professionals want to know how the tool ended up to its result…*” (AI expert, Interview 3). Another emphasized, *“…what clinicians care really about is not interpretability, but whether it has value and whether there is clinical evidence showing that this really delivers what it promises to deliver*” (AI expert, Interview 5).

All HCPs emphasized the importance of understanding how AI systems reach specific decisions, while noting that full technical details are unnecessary. One participant highlighted the challenge of the “black box” problem in neural networks, stating, “*... in terms of classification it would be very beneficial to have some sort of presentation on the exact parameters that affected the classification. If AI experts could explain the black box of neural networks that would be a holy grail of medical science*” (HCP, Interview 9).

#### Subdomain: Knowledge to use it

The theme “agreeing the scope of use”, was considered highly important by AI experts and was emphasized repeatedly. They noted that HCPs need clarity on the scope of use of AI tools, while understanding the underlying model logic is not necessary. One participant stressed the importance of training HCPs on this scope, whereas another highlighted challenges in providing such training. As one AI expert explained, “*To ensure understanding when implementing new tool, cooperation is the key…*” (AI expert, Interviewe 3) while another one stated, “…*So this is fundamental and really difficult to get everyone trained and understand this*” (AI expert, Interview 4).

Three HCPs emphasized the importance of clearly defining the scope of use for AI tools. However, two participants pointed to insufficient training from AI experts and difficulties in educating HCPs. One noted, “*...I have experienced in a project related to AI based MRI image reconstruction, that testing and responsibility for implementation was at the responsibility of the hospital, support was not provided by the company providing the AI solution*” (HCP, Interview 8). Another added, “In my experience, AI experts are unable to teach HCPs what exactly AI does and does not do...” (HCP, Interview 9).

#### Subdomain: Material properties

In relation to the theme “usability” several AI experts emphasized that ease of use matters more than extensive functionality, warning that overly complex tools risk failure. As one participant noted, “*Therefore, I believe it is much more important to create solutions that are as easy to use as possible…*” (AI expert, Interview 2). In contrast, one expert stressed that both usability and functionality are equally critical, while another pointed out that security requirements can sometimes hinder user-friendliness, explaining, “*…Well, once we have a very high standards, which is absolutely very good and it need to be there… then from the developer perspective the security equals or against the usability. Sometimes may be a tackle or fight even.*” (AI expert, Interview 1).

While two HCPs considered ease of use and functionality equally important, two others emphasized ease of use, with one also pointing to a lack of attention from AI experts. “*…in my experience software tools may even be abandoned if the ease of use is poor. It may be that AI experts and developers do not pay sufficient amount of attention to ease of use…*” (HCP, Interview 8).

### Domain: Value proposition

#### Subdomain: Demand-side value

The theme “healthcare professionals’ requirements”, identified through inductive analysis, was assigned to the NASSS subdomain of demand-side value. AI experts stressed the need to address HCPs’ requirements, though some noted misunderstandings and limited attention to demand-side concerns. One participant highlighted the economic value of AI tools alongside functional needs: “*You cannot ask healthcare professionals what kind of AI solutions could help, because they don’t know…they don’t understand what kind of things can be solved with AI*” (AI expert, Interview 4). All experts agreed that HCPs expect AI to take over routine tasks to allow more patient time, while some also emphasized decision-support algorithms: “*I think they are looking for tools that save their time to meet patients, less time spent with computers. They also expect that AI could digest the massive amount of data and bring suggestion…*” (AI expert, Interview 3).

Regarding “healthcare professionals’ requirements”, three HCPs agreed that AI should handle routine tasks, allowing them to focus on patient care. One highlighted radiology as a key area: “*The biggest expectation probably is to reduce load of radiologists to review radiological studies…and hence focus personnel resources to more challenging cases*” (HCP, Interview 8). Another stressed the need for tools with proven clinical benefit: *“…repetitive work is probably decreased but improved patient outcome has not yet been demonstrated in scientific peer-reviewed work*” (HCP, Interview 9). Additionally, two participants emphasized communication, noting that AI experts often lack insight into healthcare needs: “*AI experts do not necessarily understand the exact tools that I need and therefore the products are not as interesting as they could be*” (HCP, Interview 9).

In the subdomain of “demand-side value”, the theme “perceived value” also emerged from inductive analysis. All AI experts emphasized the importance of understanding the benefits of AI, noting that such awareness supports acceptance and strengthens collaboration. One of them explained, “*Healthcare professionals are too busy everywhere all the time but on the other hand, if they really expect to get some help from these kind of tools then they need to allocate some resources …*” (AI expert, Interview 4).

Regarding “perceived value”, only one HCP raised this factor, emphasizing its role in cooperation and acceptance of change. As the participant explained, *“…AI experts must demonstrate monetary benefit, reduced image acquisition time, reduced mortality or some other important outcome for truly selling the product”* (HCP, Interview 9).

### Domain: Adopters

#### Subdomain: Tools redefine staff roles

This subdomain was emphasized by several AI experts without a special theme, mentioning that cooperation, perceived value, and usability are important in redefining the roles of HCPs without disrupting their work. As mentioned by one AI expert, *“…To avoid disrupting their work, ease of use and usability are crucial. Ongoing interaction and collaboration are essential throughout the process*” (AI expert, Interview 2).

Two HCPs felt that AI tools supported their tasks without changing their roles. Another noted that while many providers are open to role reshaping, some remain protective, emphasizing the importance of communication between AI experts and HCPs, explaining, “*I am very open to reshaping the role of professional… I also work closely with radiologists; it would be very important to interview medical experts for this…In my work I find that other doctors are more interested in reshaping the role than others. Some are protective…”* (HCP, Interview 8). One participant highlighted concerns about professional replacement, mentioning, “*I expect AI tools to speed up my workflow but not completely replacing my work in any significant way in the near future*” (HCP, Interview 9).

### Domain: Organization

#### Subdomain: Extent of change needed to organizational routines

Regarding the theme “Fitting the tool within current practices”, three of the AI experts highlighted the importance of integrating AI tools into existing practices. They noted that smooth implementation requires involving users already during the development stage. As stated by one AI expert, *“In healthcare, workflows are often complex and well-established, so smoothly integrating a new tool into existing practices is essential. If an AI solution requires significant changes or disrupts current workflows, its adoption may face resistance and remain limited”* (AI expert, Interview 2).

Out of three HCPs discussing this factor, two of them did not report problems in this area noting, “*We are used to changes. In the medical field, the work environment changes all the time*” (HCP, Interview 7). However, one emphasized that perceived value and benefits is important for accepting change and fostering cooperation explaining, “*Also, we do not see any need to implement AI if it does not hold significant benefit. As such, our department does not change its routines or workflow for AI implementation*” (HCP, Interview 9).

### Domain: Wider context

#### Regulatory and legal issues

In this subdomain, regarding the theme “deciding who is responsible”, most AI experts emphasized that HCPs hold the final responsibility for clinical decisions, as defined by regulations. Some added that this also depends on AI experts clearly defining and training the scope of use: well-informed HCPs are more likely to trust AI and accept accountability, while unclear training on the scope of use may shift responsibility back to developers. As explained by one AI expert “*Also, the regulation of course is giving some limits here, so most of the time it has to be really clear that the professional is there one who is responsible. Model tries to help but doesn’t always work. Then it’s a manufacturer who is quite a lot responsible of those that how it’s defined that in what cases you should use this …*” (AI expert, Interview 4)”.

Most HCPs also believed they should remain responsible for final decisions, citing limited trust in AI. As one participant noted, “I would love that the AI would do all the dirty work…we cannot yet trust what it decides…humans have brains which are much more complicated, and we can make the decision making and learn all day” (HCP, Interview 7).

#### Subdomain: Professional bodies

The theme “Lack of understanding between professional groups” was highlighted by all AI experts, noting that HCPs often lack awareness of AI’s capabilities, while AI experts struggle to understand healthcare needs. Interdisciplinary experts were seen as essential to bridge this gap. One participant added that insufficient understanding on the developers’ side can also foster HCP distrust: “*Yes, I believe there is a significant lack of understanding between healthcare professionals and AI experts in the AI implementation process…. There is definitely a need for interdisciplinary experts*” (AI expert, Interview 2).

Two HCPs felt there was no misunderstanding with AI experts and therefore no need for an interdisciplinary role. As one explained, “*I don’t think there needs to be interdisciplinary experts…the experts understand very well the medical experts’ ideas, even if they don’t know the substance*” (HCP, Interview 7). In contrast, another participant believed that especially in Finland, communication is lacking and that an intermediary would be valuable: “*At least in Finland, I don’t think there is enough discussion between AI experts developing solutions and healthcare professionals. Experts filling this gap would be welcome…*” (HCP, Interview 8).

#### Subdomain: Interorganizational networking

The factor of interorganizational networking, identified through inductive analysis without a special theme, aligns with the corresponding NASSS subdomain. AI experts emphasized the need for cooperation with HCPs, starting in development and continuing through implementation. Some noted HCPs’ limited availability due to heavy workloads but believed that once convinced of a tool’s value, they would engage actively. As one AI expert stated, “*Unfortunately at the moment healthcare professionals are really busy because of the shortage of the staff …but my experience is that if they believe in AI tool they are motivated to cooperate, but we as AI experts do need to be flexible as well*” (AI expert, Interview 3).

All HCPs agreed on the need for close cooperation with AI experts during both development and implementation. One participant described the positive impact of early involvement: “*…it’s been really, really important that there’s been medical experience experts in the group from the beginning and I have been in hackathon where there’s been like many different groups making AI tools and you could see from the start that some of them didn’t have any medical experts with them*” (HCP, Interview 7). However, views differed on collaboration with commercial AI experts. One HCP valued such cooperation: “…*The AI expert care nowadays very much about the cooperation – especially with firms with commercial AI software…*” (HCP, Interview 6). In contrast, another expressed reluctance: “*The benefits are not distributed to facilities helping to develop these tools. As such, I have no interest of cooperating with companies in this manner*” (HCP, Interview 9).

### Domain: Embedding and adaptation over time

#### Subdomain: Organizational resilience

This subdomain was identified as important by most AI experts without a specific theme. They stressed the need for close cooperation between HCPs and AI specialists in both adapting and developing AI tools. One participant shared challenges in cooperation, noting that collaboration is closely tied to change management. As one AI expert stated, “*Healthcare professionals know the specific characteristics of the field, and only they can identify the challenges the service should address. They must also be involved in testing and development to ensure that the service becomes as useful as possible for their needs.*” (AI expert, Interview 2).

Most HCPs also emphasized the importance of cooperation with AI experts during the adaptation process. As one participant explained, “*Cooperation between healthcare professionals and AI experts is very important…I would imagine that AI experts would care about adaptation and cooperation*” (HCP, Interview 8).

### Most highlighted factors

In response to the free-text question on the most important factors, two AI experts highlighted demand-side value. Other factors mentioned once included interorganizational cooperation, transparency and trust, lack of understanding between professional groups, and privacy and security.

In response to this question, two HCPs identified transparency and trust as the most important factors. Other factors mentioned once included usability, interorganizational cooperation, and demand-side value.

### Patterns

The patterns identified HCPs and AI experts are summarized in Table 2 to support the development of a framework for improving cooperation between the two groups.

**Table 2.** Key patterns emerging from the interviews.

Pattern	Number of participants mentioning
Transparency in AI tools fosters trust	5 AI experts and 1 HCP
Considering demand-side value including actual and perceived value of HCPs leads to acceptance and collaboration	5 AI experts and 1 HCP
Early involvement of HCPs supports identification of demand-side value	2 AI experts and 2 HCPs
Limited understanding between AI experts and HCPs hinders recognition of demand-side value	2 AI experts and 1 HCP
Training HCPs improves understanding of the scope of use	2 AI experts and 1 HCP
Lack of mutual understanding contributes to distrust	2 AI experts
Agreement on scope of use clarifies responsibilities	2 AI experts
Collaboration between AI experts and HCPs builds trust	1 AI expert
Understanding scope of use strengthens HCPs’ trust	1 AI expert
Collaboration helps HCPs better understand scope of use	1 AI expert
Trust reduces resistance among HCPs	1 AI expert

## Discussion

### Answer to research questions and critical interpretation

This study identified both alignments and gaps between AI experts and HCPs, analyzed through the NASSS framework using thematic analysis. In the domain of Condition, both groups agreed that AI tools still require significant improvement to manage complex cases, incorporate socio-cultural factors, and address comorbidities. The absence of major gaps in this domain suggests that limitations in this domain are broadly recognized, and while not affecting the collaboration as a gap, can restrict the AI adoption in the healthcare sector.

Transparency, identified as one of the most important factors by both AI experts and HCPs, was linked to trust in six interviews. Both AI experts and HCPs emphasized that transparent communication of AI outputs is essential for building trust. This reflects earlier findings, such as Reddy,^32^ who argued that the complexity of generative AI undermines trust when transparency is lacking. Similar concerns were noted by Bhavaraju^48^ and Alanazi,^28^ who highlighted trust as a central barrier to adoption for healthcare providers, while Vinh Vo et al.^50^ reported transparency as a top priority for stakeholders. Hogg et al.^18^ further observed that the “Black Box” nature of non–rule-based AI produced mixed reactions, with some HCPs viewing responsibility as their own to improve understanding, while others placed it on developers to provide more interpretable metrics. This indicates that transparency functions as a foundation for trust and AI adoption in healthcare, primarily by influencing healthcare professionals’ willingness to engage in collaboration.

Interorganizational cooperation and early involvement of HCPs were viewed by both groups as essential and among the most important factors, for aligning AI tools with clinical needs and facilitating integration. Vinh Vo et al.,^50^ likewise emphasized clinician participation in the development process of artificial intelligence in healthcare. Weinert et al.^41^ also reported that collaboration with clinicians helps define shared goals, identify useful algorithms, and assess usability in practice, which is in line with the pattern identified in this study. Despite agreement on its importance, perspectives diverged: AI experts questioned HCPs’ willingness to engage, whereas HCPs expressed differing views on working with commercial versus academic partners, with some favoring industry collaboration and others preferring internal expertise. Similar tensions are reflected in prior studies. Laï et al.^51^ noted that many HCPs see industry-developed AI as a way to save time and streamline tasks, while Hogg et al.^18^ observed that effective cooperation requires both clinical and technical expertise but is often hindered by the workload demands of HCPs. This suggests that although collaboration is valued by both groups, limited trust and the absence of clear mechanisms for effective collaboration can hinder smooth implementation.

Demand-side value, understood here as both actual and perceived value for HCPs, was of the most important factors and strongly emphasized by both AI experts and HCPs for developing effective AI solutions. Bhavaraju^52^ similarly argued that AI development should begin with careful evaluation of use cases and a clear understanding of user needs. This study found that greater perceived value encouraged collaboration, echoing Hogg et al.,^18^ who reported that HCPs engage more when they recognize value in context. Petersson et al.^13^ also concluded that demonstrating tangible benefits for daily tasks is crucial to enhancing HCPs’ motivation and engagement. Despite this alignment, notable gaps emerged. AI experts largely viewed AI as a means to automate routine work, whereas HCPs also expected decision-support capabilities and transparent outputs. Some experts perceived HCPs as unclear about their own needs, while HCPs criticized developers for a limited understanding of clinical processes and excessive focus on financial returns. Similar findings appear in prior studies: Ahmed et al.^53^ and Swed et al.^54^ reported that only 23% and 27% of physicians, respectively, were aware of AI use in healthcare, and Laï et al.^51^ noted a gap between scientific progress and the exaggerated claims promoted in the media.

Both HCPs and AI experts stressed the importance of clearly defining the scope of use to set expectations and clarify responsibilities in AI implementation. Prior studies have likewise highlighted the need for HCP training, especially on the scope of use.^13,32,50,55^ In this study, however, some HCPs reported insufficient support from AI experts, with training responsibilities shifted to hospitals, while one AI expert pointed to difficulties in training HCPs. This reflects gaps in cooperation and unclear role specification, which is also found in the domain of interorganizational cooperation. Similar concerns were reported by Hogg et al.,^18^ who observed cases where AI tools were abandoned due to limited understanding, and by Laï et al.,^51^ who noted that industry professionals often underestimated the importance of physicians fully understanding how AI tools operate, sometimes suggesting only superficial training.

Ease of use was highlighted by both groups as critical, since intuitive tools are more likely to be adopted in busy healthcare settings. Petersson et al.^13^ similarly noted that healthcare leaders expect AI systems to be simple and require minimal training. Weinert et al.^41^ also found that usability was among the most frequently identified subdomains in AI studies. In this study, some HCPs reported that usability was often overlooked during development, while one AI expert argued that functional value could compensate for limited usability. While ease of use was not raised by any AI experts as one of the most important factors in this study, Hogg et al.^18^ reported that developers themselves considered usability and accessibility to influence adoption more strongly than performance. These gaps suggest that limited interaction and the absence of shared goals between the groups may have contributed to differing expectations regarding ease of use.

Staff roles were not seen as a major source of resistance, as both HCPs and AI experts emphasized that changes are acceptable when supported by cooperation, usability, and value. One HCP raised concerns about replacement by AI, but most felt their primary roles remained intact and noted that adapting to change is part of their profession. This aligns with Laï et al.,^51^ who reported that physicians are generally open to reevaluating roles as long as they remain central to patient care, their primary goal. AI experts also stressed smooth integration to avoid disrupting clinical work, echoing Hogg et al.,^18^ who found that gradual, HCP-led transfers of responsibility support successful AI adoption.

On the issue of mutual understanding, most AI experts in this study pointed to a communication gap with HCPs and suggested interdisciplinary experts as a bridge. In contrast, some HCPs felt no such misunderstanding existed, highlighting divergent perceptions of collaboration. Moreover, while one AI expert emphasized a lack of understanding as the most important factor, no other HCPs had the same idea. Thenral and Annamalai^27^ also identified the shortage of interdisciplinary experts as a barrier to developing deployable AI-enabled telepsychiatry solutions. Petersson et al.^13^ noted that healthcare leaders viewed communication across professional groups as a major obstacle, as they do not share the same professional language, stressing the need to adapt training to prepare future HCPs for digital technologies. He et al.^24^ and Vinh Vo et al.^50^ also argued the importance of investment in training and educational initiatives for HCPs, emphasizing that HCPs require education on both the benefits and limitations of AI with a suggestion that curricula incorporate health informatics, computer science, and statistics. 

On the issue of responsibility, both groups in this study agreed that final accountability should rest with HCPs, as they remain the ultimate decision makers, while AI experts also stressed their duty to provide adequate training on the scope of use. However, a gap also appeared regarding AI autonomy: AI experts felt HCPs preferred to retain final decision-making authority in all cases, whereas some HCPs welcomed full automatization in specific areas such as radiology. Laï et al.^51^ similarly identified responsibility as a central concern, with most stakeholder groups—physicians, industry partners, institutions, and independent individuals—reluctant to accept liability for patient harm, while Physicians were willing to assume responsibility only if they had a clear understanding of how the tool reached its conclusions, which aligns with the findings of this study. This tension reflects an unresolved boundary between human oversight and automation, suggesting that acceptable levels of AI autonomy may be context-specific rather than universally defined.

On data quality and security, AI experts in this study generally did not highlight major issues, whereas HCPs raised concerns about training with external datasets, as well as data privacy and storage, which reduced trust and willingness to cooperate. Similar concerns are reflected in previous research on privacy and security risks.^27,28,32,52^ Petersson et al.^13^ also noted challenges in defining personal data within regulatory frameworks and the risk of reidentification. Data quality training was another recurring issue. Bhavaraju^52^ emphasized that AI performance depends on careful collection, curation, and use of training data, while Laï et al.^51^ reported that developers often face difficulties accessing clinical data for algorithm training. In this study, one HCP mentioned reluctance to share data due to privacy concerns, echoing Hogg et al.,^18^ who found that developers frequently encountered resistance from healthcare organizations and patients unwilling to grant access to sensitive data.

Finally, this study contributes to the sustainable value proposition of AI in healthcare by emphasizing responsible adoption. De Andreis et al.^56^ noted that transparency, accountability, and effective data management are central to ethical and sustainable AI use, all of which were highlighted in this study. As AI adoption expands, concerns about its environmental impact also increase.^10^ This underscores the need to prioritize high-impact applications that address real healthcare demands, consistent with the results of this study in demand-side value.

### Implications for the NASSS framework

This study showed that the NASSS framework is a useful tool for evaluating collaboration between different stakeholders throughout technology implementation. The comprehensive domains and subdomains of the framework support a systematic examination of how differing perspectives on each factor, between collaborating groups, can influence cooperation and affect implementation.

Moreover, through inductive analysis, this study added healthcare professionals’ values to the demand-side value domain, which previously focused on patients’ values in the original framework. This study showed the importance of considering the values of all the stakeholders for better collaboration and can be considered in future research. In addition, perceived value emerged through inductive analysis and was added to the demand-side value as a theme. Alongside actual delivered value, perceived value was found to be an influential factor in implementation and was extensively emphasized by participants, which is in line with the TAM framework.^36^ Together, these additions demonstrate the flexibility of the NASSS framework and support extending its use to evaluate collaboration between stakeholders.

### Proposed framework

**Figure 3.**
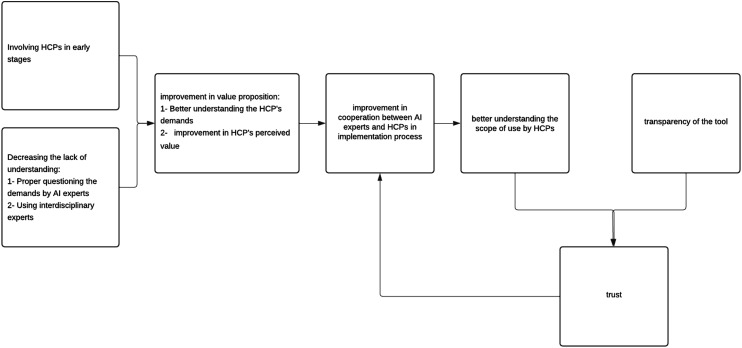
Proposed framework to enhance collaboration between AI experts and healthcare professionals in the implementation of AI in healthcare.

Rather than considering each NASSS subdomain or theme in isolation, this study goes beyond individual factors by identifying common patterns across interviews and examining their interactions, which informed the development of the proposed framework. The framework (Figure 3) was derived from the shared patterns identified in Table 3 and aims to strengthen collaboration between healthcare professionals (HCPs) and AI experts by focusing on values shared by both groups and illustrating the process of initiating and reinforcing collaboration. It highlights the importance of involving HCPs early in tool development, improving communication with AI experts, and using interdisciplinary experts to better identify demand-side value. This leads to improvement in value proposition, including perceived value among HCPs as they understand tool advantages and find their influence on the final product, which in turn strengthens cooperation during implementation. This close collaboration helps with training HCPs and clarification on the scope of use and together with transparency in the communication of results, fosters trust and further supports cooperation during adoption. This framework is grounded in the study data and requires validation with a larger sample of HCPs and AI experts.

### Practical implications

The findings of this study offer several implications for the successful and sustainable implementation of AI in healthcare. Early and continuous involvement of HCPs is essential to align AI tools with clinical workflows and ensure their relevance in practice. Structured collaboration mechanisms, supported by interdisciplinary expertise, can bridge the communication gap between clinicians and AI developers and strengthen mutual trust.

The need for interdisciplinary expertise, emphasized in this study, also highlights implications for workforce development and education policy. This requires targeted training solutions that equip HCPs with foundational AI literacy and provide AI experts with greater exposure to clinical contexts. Including interdisciplinary competencies in healthcare education, professional development programs, and organizational training strategies are among the solutions that can be considered to strengthen interdisciplinary expertise.

Equally important is a focus on demand-side and perceived value. AI tools should be designed to reduce routine workload while also supporting decision-making, thereby demonstrating tangible benefits for providers. Regular feedback loops between HCPs and developers can enhance usability and foster greater engagement.

Transparency and a clearly defined scope of use are critical to fostering trust. Providing interpretable outputs, clarifying responsibilities, and delivering structured training enable HCPs to use AI tools effectively and safely. At the same time, usability must be prioritized to ensure that tools integrate seamlessly into demanding clinical environments.

Finally, concerns regarding data privacy, security, and quality need to be addressed. Transparent practices, compliance with regulations, and validation on local datasets are necessary to reduce resistance and encourage data sharing. Developers should also be realistic about the current limitations of AI in managing comorbidities and complex conditions, avoiding overstated claims. Taken together, these findings provide a roadmap for managers and decision makers to foster trust, strengthen collaboration, and ensure that AI systems deliver real value in clinical practice.

### Limitations and future research

This study has several limitations that should be considered when interpreting its findings. The sample size was small and limited to Finland, which may restrict the generalizability of the results and the proposed framework. While the qualitative method and precise selection of participants with substantial experience in the field provided in-depth insights, a larger and more diverse sample could have captured a broader range of perspectives. Time constraints during interviews also limited exploration of some themes, particularly more complex aspects of AI implementation. In addition, demographic data such as age, gender, and years of experience were not collected; including such information in future studies could provide a richer understanding of how personal characteristics shape perceptions of AI adoption.

Future research should address these limitations by expanding the number of participants and combining qualitative and quantitative approaches. Longer or repeated interviews, alongside focus groups, could yield more nuanced insights and facilitate dialogue between HCPs and AI experts. Further studies with more samples are also needed to validate and refine the proposed framework across diverse healthcare settings and stakeholder groups, which would help assess its applicability and strengthen its relevance for guiding effective AI implementation.

## Conclusion

This study explored the perspectives of HCPs and AI experts on the success factors for implementing AI in healthcare, applying the NASSS framework to capture both alignments and gaps. Transparency and trust, importance of usability, understanding the scope of use by HCPs, early involvement of HCPs, responsibility, and redefining the roles emerged as shared priorities, while differences were observed in collaboration preferences, lack of understanding, the need for interdisciplinary experts, expectations of demand-side value, views on responsibility, lack of training, data quality, and privacy.

The proposed framework emphasizes early collaboration, clear communication, training, and interdisciplinary support as key strategies to strengthen trust and cooperation. It also promotes the sustainability of AI integration in healthcare by highlighting transparency, accountability, data privacy, and data quality, while emphasizing the importance of understanding demand-side value to ensure that resources are directed toward applications delivering meaningful clinical impact.

Taken together, this study contributes both theoretical insights into stakeholder collaboration and practical guidance for managers and developers seeking to integrate AI responsibly and sustainably into healthcare practice.

## Supplemental material


Supplemental Material - Bridging perspectives: Success factors for AI implementation in healthcare from healthcare professionals and AI experts
Supplemental Material for Bridging perspectives: Success factors for AI implementation in healthcare from healthcare professionals and AI experts by Zohreh Yousefi Dahka and Timo Koivumäki in Digital Health.

Supplemental Material - Bridging perspectives: Success factors for AI implementation in healthcare from healthcare professionals and AI experts
Supplemental Material for Bridging perspectives: Success factors for AI implementation in healthcare from healthcare professionals and AI experts by Zohreh Yousefi Dahka and Timo Koivumäki in Digital Health.

## Data Availability

The interview data cannot be shared publicly, as participants were not informed that their data would be made openly available, in accordance with GDPR requirements. However, the metadata describing the dataset has been published in Fairdata.fi and is cited within the text.*

## References

[1] Newlands R Bruhn H Díaz MR , et al. A stakeholder analysis to prepare for real-world evaluation of integrating artificial intelligent algorithms into breast screening (PREP-AIR study): A qualitative study using the WHO guide. BMC Health Serv Res 2024; 24: 569. 10.1186/s12913-024-10926-z 38698386 PMC11067265

[2] Ramalingam A Karunamurthy A Pavithra B . Impact of artificial intelligence on healthcare: A review of current applications and future possibilities. Quing Int J Innov Res Sci Eng 2023; 2: 37–49. 10.54368/qijirse.2.2.0005

[3] Pavli A Theodoridou M Maltezou HC . Post-COVID syndrome: Incidence, clinical spectrum, and challenges for primary healthcare professionals. Arch Med Res 2021; 52: 575–581. 10.1016/j.arcmed.2021.03.010 33962805 PMC8093949

[4] Myllylä J . Artificial intelligence applications in Finnish healthcare. Master’s thesis, Satakunta University of Applied Sciences, 2023. Available from. https://urn.fi/urn:nbn:fi:amk-202305017031

[5] Gilson A Safranek CW Huang T , et al. How does ChatGPT perform on the United States Medical Licensing Examination? The implications of large language models for medical education and knowledge assessment. JMIR Med Educ 2023; 9: e45312. 10.2196/45312 36753318 PMC9947764

[6] Aminizadeh S Heidari A Dehghan M , et al. Opportunities and challenges of artificial intelligence and distributed systems to improve the quality of healthcare service. Artif Intell Med 2024; 149: 102779. 10.1016/j.artmed.2024.102779 38462281

[7] Davenport T Kalakota R . The potential for artificial intelligence in healthcare. Future Healthc J 2019; 6(2): 94–98. 10.7861/futurehosp.6-2-94 PMC6616181 31363513

[8] Malyshev V Lipskyi Y Kovalenko V , et al. Assessment of the global artificial intelligence market in healthcare. Technol Audit Prod Reserves. 2024;6(4(80)):62–70. 10.15587/2706-5448.2024.316451

[9] Coiera EW Verspoor K Hansen DP . We need to chat about artificial intelligence. Med J Aust 2023; 219: 98–100. 10.5694/mja2.51992 37302124 PMC10952508

[10] Ueda D Walston SL Fujita S , et al. Climate change and artificial intelligence in healthcare: Review and recommendations towards a sustainable future. Diagn Interv Imaging 2024; 105(11): 453–459. 10.1016/j.diii.2024.07.005 38918123

[11] Novillo-Ortiz D de Fátima Marin H Saigí-Rubió F . The role of digital health to support the achievement of the Sustainable Development Goals (SDGs). World Health Organization, 2018. 10.1016/j.ijmedinf.2018.03.011 29602629

[12] Alhussain G Kelly A O’Flaherty EI , et al. Emerging role of artificial intelligence in global health care. Health Policy Technol 2022; 11(3): 100661. 10.1016/j.hlpt.2022.100661 35991006 PMC9374598

[13] Petersson L Larsson I Nygren JM , et al. Challenges to implementing artificial intelligence in healthcare: A qualitative interview study with healthcare leaders in Sweden. BMC Health Serv Res 2022; 22: 850. 10.1186/s12913-022-08215-8 35778736 PMC9250210

[14] Huma S Alhamzah FA Nohman K , et al. Digital technologies in healthcare: a systematic review and bibliometric analysis. Int J Online Biomed Eng 2022; 18(8): 34–48.

[15] Rajpurkar P Chen E Banerjee O , et al. AI in health and medicine. Nat Med 2022; 28: 31–38. 10.1038/s41591-021-01614-0 35058619

[16] Svedberg P Reed J Nilsen P , et al. Toward successful implementation of artificial intelligence in healthcare practice: Protocol for a research program. JMIR Res Protoc 2022; 11(3): e34920. 10.2196/34920 35262500 PMC8943554

[17] Knopp MI Warm EJ Weber D , et al. AI-enabled medical education: Threads of change, promising futures, and risky realities across four potential future worlds. JMIR Med Educ 2023; 9: e50373. 10.2196/50373 38145471 PMC10786199

[18] Hogg H Al-Zubaidy M Technology Enhanced Macular Services Study Reference Group , et al. Stakeholder perspectives of clinical artificial intelligence implementation: Systematic review of qualitative evidence. J Med Internet Res 2023; 25: e39742. 10.2196/39742 36626192 PMC9875023

[19] Yousefi Dahka Z Koivumäki T . AI implementation in healthcare: key success factors from HCPs and AI experts. J Bus Models Forthcoming. Special issue. 10.1177/20552076261437277 PMC13018691 41907364

[20] Faxvaag A Reponen J Hardardottir GA , et al. Towards accountable e-health policies in the Nordic countries. Stud Health Technol Inform 2024; 316: 339–343. 10.3233/SHTI240413 39176742

[21] Wolff J Pauling J Keck A , et al. Success factors of artificial intelligence implementation in healthcare. Front Digit Health 2021; 3: 594971. 10.3389/fdgth.2021.594971 34713083 PMC8521923

[22] Greenhalgh T Abimbola S . The NASSS framework: A synthesis of multiple theories of technology implementation. Stud Health Technol Inform 2019; 263: 193–204. 10.3233/SHTI190123 31411163

[23] Dicuonzo G Donofrio F Fusco A , et al. Healthcare system: Moving forward with artificial intelligence. Technovation 2023; 120: 102510. 10.1016/j.technovation.2023.102510

[24] He J Baxter SL Xu J , et al. The practical implementation of artificial intelligence technologies in medicine. Nat Med 2019; 25(1): 30–36. 10.1038/s41591-018-0307-0 30617336 PMC6995276

[25] Gama F Tyskbo D Nygren J , et al. Implementation frameworks for artificial intelligence translation into healthcare practice: Scoping review. J Med Internet Res 2022; 24(1): e32215. 10.2196/32215 35084349 PMC8832266

[26] Elish MC . The stakes of uncertainty: developing and integrating machine learning in clinical care. Ethnogr Prax Ind Conf Proc 2018; 2018(1): 364–380. 10.1111/1559-8918.2018.01213

[27] Thenral M Annamalai A . Challenges of building, deploying, and using AI-enabled telepsychiatry platforms for clinical practice among urban Indians: a qualitative study. Indian J Psychol Med 2020; 42(5): 445–451. 10.1177/0253717620949628 34385728 PMC8327861

[28] Alanazi A . Clinicians’ views on using artificial intelligence in healthcare: Opportunities, challenges, and beyond. Cureus 2023; 15(9): e45255. 10.7759/cureus.45255 37842420 PMC10576621

[29] Alami H Lehoux P Papoutsi C , et al. Understanding the integration of artificial intelligence in healthcare organisations and systems through the NASSS framework: A qualitative study in a leading Canadian academic centre. BMC Health Serv Res 2024; 24: 701. 10.1186/s12913-024-11112-x 38831298 PMC11149257

[30] Wubineh BZ Deriba FG Woldeyohannis MM . Exploring the opportunities and challenges of implementing artificial intelligence in healthcare: A systematic literature review. Urol Oncol 2024; 42(3): 48–56. 10.1016/j.urolonc.2023.11.019 38101991

[31] Greenhalgh T Wherton J Papoutsi C , et al. Beyond adoption: A new framework for theorizing and evaluating nonadoption, abandonment, and challenges to the scale-up, spread, and sustainability of health and care technologies. J Med Internet Res 2017; 19(11): e367. 10.2196/jmir.8775 29092808 PMC5688245

[32] Reddy S . Generative AI in healthcare: An implementation science informed translational path on application, integration, and governance. Implement Sci 2024; 19: 27. 10.1186/s13012-024-01357-9 38491544 PMC10941464

[33] Nair M Andersson J Nygren J , et al. Barriers and enablers for implementation of an artificial intelligence–based decision support tool to reduce the risk of readmission of patients with heart failure: Stakeholder interviews. JMIR Form Res 2023; 7: e47335. 10.2196/47335 37610799 PMC10483295

[34] Strohm L Hehakaya C Ranschaert ER , et al. Implementation of artificial intelligence (AI) applications in radiology: Hindering and facilitating factors. Eur Radiol 2020; 30(11): 5525–5532. 10.1007/s00330-020-06946-y 32458173 PMC7476917

[35] Koebe P . How digital technologies and AI contribute to achieving the health-related SDGs. Int J Inf Manag Data Insights 2025; 5(1): 100298. 10.1016/j.ijimei.2025.100298

[36] Davis FD . Perceived usefulness, perceived ease of use, and user acceptance of information technology. MIS Q 1989; 13(3): 319–340. 10.2307/249008

[37] Venkatesh V Morris MG Davis GB , et al. User acceptance of information technology: toward a unified view. MIS Q 2003; 27(3): 425–478. 10.2307/30036540

[38] Feldstein AC Glasgow RE . A practical, robust implementation and sustainability model (PRISM) for integrating research findings into practice. Jt Comm J Qual Patient Saf 2008; 34(4): 228–243. 10.1016/S1553-7250(08)34030-6 18468362

[39] Glasgow RE Vogt TM Boles SM . Evaluating the public health impact of health promotion interventions: The RE-AIM framework. Am J Public Health 1999; 89(9): 1322–1327. 10.2105/ajph.89.9.1322 10474547 PMC1508772

[40] Damschroder LJ Aron DC Keith RE , et al. Fostering implementation of health services research findings into practice: A consolidated framework for advancing implementation science. Implement Sci 2009; 4: 50. 10.1186/1748-5908-4-50 19664226 PMC2736161

[41] Weinert L Klass M Schneider G , et al. Exploring stakeholder requirements to enable research and development of artificial intelligence algorithms in a hospital-based generic infrastructure: Results of a multistep mixed-methods study. JMIR Form Res 2023; 7: e43958. 10.2196/43958 37071450 PMC10155093

[42] Kelly SE . Qualitative interviewing techniques and styles. In: Bourgeault I Dingwall R De Vries R (eds). The SAGE handbook of qualitative methods in health research. Sage Publications, 2010, pp. 307–326. 10.4135/9781446268247.n17

[43] Yousefi Dahka Z . Semi-structured interviews with healthcare professionals and AI experts on AI implementation in healthcare (Finland, 2025) [dataset]. Version 1. University of Oulu, Martti Ahtisaari Instituutti, 2024. Available from. https://urn.fi/urn:nbn:fi:fd-45e1dcc6-7321-3e7b-a593-b783abc18efe

[44] Finnish National Board on Research Integrity TENK . The Finnish Code of Conduct for Research Integrity and Procedures for Handling Alleged Violations of Research Integrity in Finland 2023. Finnish National Board on Research Integrity TENK, 2023. [cited 2026 Feb 9]. Available from. https://tenk.fi/sites/default/files/2023-05/RI_Guidelines_2023.pdf

[45] European Parliament and Council of the European Union . Regulation (EU) 2016/679 of the European Parliament and of the Council of 27 April 2016 on the protection of natural persons with regard to the processing of personal data and on the free movement of such data (General Data Protection Regulation). Off J Eur Union 2016; L119: 1–88.

[46] Braun V Clarke V . Using thematic analysis in psychology. Qual Res Psychol 2006; 3: 77–101. 10.1191/1478088706qp063oa

[47] Clarke V Braun V . Teaching thematic analysis: overcoming challenges and developing strategies for effective learning. The Psychologist 2013; 26(2): 120–123.

[48] Nenonen S Brodie RJ Storbacka K , et al. Theorizing with managers: How to achieve both academic rigor and practical relevance? Eur J Mark 2017; 51(7–8): 1130–1152. 10.1108/ejm-03-2017-0171

[49] Thompson J . A guide to abductive thematic analysis. Qual Rep 2022; 27(5): 1410–1421. 10.46743/2160-3715/2022.5340

[50] Vinh Vo G Chen Y Aquino YSJ , et al. Multi-stakeholder preferences for the use of artificial intelligence in healthcare: A systematic review and thematic analysis. Soc Sci Med 2023; 338: 116357. 10.1016/j.socscimed.2023.116357 37949020

[51] Laï MC Brian M Mamzer MF . Perceptions of artificial intelligence in healthcare: Findings from a qualitative survey study among actors in France. J Transl Med 2020; 18(1): 14. 10.1186/s12967-019-02204-y 31918710 PMC6953249

[52] Bhavaraju SR . Artificial intelligence in healthcare: doctor as a stakeholder. IntechOpen, 2023. 10.5772/intechopen.111490

[53] Ahmed Z Bhinder KK Tariq A , et al. Knowledge, attitude, and practice of artificial intelligence among doctors and medical students in Pakistan: A cross-sectional online survey. Ann Med Surg (Lond) 2022; 76: 103493. 10.1016/j.amsu.2022.103493 35308436 PMC8928127

[54] Swed S Alibrahim H Elkalagi NKH , et al. Knowledge, attitude, and practice of artificial intelligence among doctors and medical students in Syria: A cross-sectional online survey. Front Artif Intell 2022; 5: 1011524. 10.3389/frai.2022.1011524 36248622 PMC9558737

[55] Olushola A Mart J Alao V . Implementations of artificial intelligence in health care. [Preprint]. ResearchGate 2023. 10.13140/RG.2.2.36344.62729/1

[56] De Andreis F Comite U Gallo A . Sustainable business model, artificial intelligence, and sustainable practices: a possible strategy for tomorrow. Int J Acad Res Bus Soc Sci. 2024; 14(1): 60–73. 10.6007/IJARBSS/v14-i1/20418

[57] Topooco N Fowler LA Fitzsimmons-Craft EE et al. Digital interventions to address mental health needs in colleges: Perspectives of student stakeholders. Internet Interventions 2022; 28: 100528. 10.1016/j.invent.2022.100528 35378846 PMC8976123

