# Supplementary material for: Bridging perspectives: Success factors for AI implementation in healthcare from healthcare professionals and AI experts
Source: Digit Health. 2026 Mar 25;12:20552076261437277. doi: 10.1177/20552076261437277 (PMC13018691; doi:10.1177/20552076261437277)
Supplement: Supplemental Material - Bridging perspectives: Success factors for AI implementation in healthcare from healthcare professionals and AI experts [file sj-pdf-2-dhj-10.1177_20552076261437277.pdf]

Appendix 1

Detailed theoretical framework of the current study driven from the NASSS framework

NASSS domain	NASSS subdomain	Themes and the selection reason
**Condition**<br><br>This domain is not mentioned to be important for healthcare professionals or AI experts in the study by Hogg and colleagues (2023), however this study has considered it to cover all the NASSS domains, because it is related to the relationship between	Nature of condition or illness	No special theme is considered, and themes will be found through analysis due to the extensive conditions that may exist for this subdomain.
	Comorbidities	No special theme is considered, and themes will be found through analysis due to the extensive conditions that may exist for this subdomain.
	Socio-cultural factors	No special theme is considered, and themes will be found through analysis due to the extensive conditions that may exist for this subdomain.

the two stakeholders and it is important based on literature (Nair et al., 2023; Doraiswamy et al., 2020)		
**Technology**	Material properties	"*Usability of the tool*" is considered for analyzing the factors related to the TAM model, perceived usefulness and ease of use, which also exists in the NASSS framework and are mentioned to be helpful according to Reddy (2024) for AI implementation in healthcare.
	Knowledge to use it	For this subdomain the theme "*agreeing the scope of use*" is considered due to being mentioned as an important factor based on Hogg and colleagues (2023) for both healthcare professionals and AI experts.
	Knowledge generated by it	For this domain "*Communicate meaning effectively*" is considered due to being important to healthcare professionals (Hogg

		et al., 2023). Moreover, it is directly related to the relationship between the two groups of stakeholders, and several studies have emphasized it (Laï et al., 2020; Nair et al., 2023; Newlands et al., 2024; Olushola et al., 2023; Reddy, 2024)
	Supply model	For this subdomain the theme "*Quality of the health data and guidelines used*" is considered due to being mentioned as an important factor based on Hogg and colleagues (2023) for both healthcare professionals and AI experts.
	Care pathway positioning	For this domain "*Extent of tools' independence*" is considered due to being important to healthcare professionals (Hogg et al., 2023) and being mentioned in 23 percent of the papers identified in the study. Moreover, the factor is related to the relationship between the two stakeholders.

**Value proposition**<br><br>The subdomain "Supply-side value" is ignored due to the low number of papers finding the subdomain important and the intention of the AI experts that considered this factor to be important has not been related to their relationship with healthcare professionals according to Hogg and colleagues (2023).	Demand-side value	No special theme is identified for this subdomain, and themes will be found in analysis because of the extensive requirements that exist from the demand side (healthcare professionals in this study)
**Adopters**<br><br>The subdomains "Patients" and "Relationships" are ignored due to being	Staff (role and identity)	All the three themes are included due to being important according to either healthcare professionals or AI experts based on Hogg and colleagues (2023).<br><br>• *Appetite and needs differ between staff*

related to the relationship between healthcare professionals and patients.		*groups*<br>• *Tools redefine staff roles*<br>• *Aligning with staff values*
**Organization**<br><br>The subdomains "Capacity to innovate in general", "Readiness for this technology", "Nature of adoption or funding decision", and "Work needed to plan, implement, and monitor change" are ignored due to not being related directly to the relationship between healthcare professionals and AI experts or be controlled by them.	Extent of change needed to organizational routines	In this subdomain the theme "*Fitting the tool within current practices*" is considered due to being important for both healthcare professionals and AI experts (Hogg et al., 2023).

**Wider Context**<br><br>The subdomain "Political or policy context" is ignored due to not being related to the relationship between healthcare professionals and AI experts	Professional bodies	The theme "*Lack of understanding between professional groups*" is considered due to being critical for both stakeholders according to Hogg and colleagues (2023).
	Regulatory and legal issues	Neither of the themes in this subdomain are identified from the perspective of healthcare professionals or AI experts, however the theme "*Deciding who is responsible*" is considered as it affects the relationship between the two groups and is important according to several research works (Wolff et al., 2021; Laï et al., 2020; Nair et al., 2023; Petersson et al., 2022; Thenral & Annamalai, 2020; Topooco et al., 2022)
**Embedding and Adaptation over Time**	Scope for adaptation over time	No special theme is considered, and themes will be found through analysis
	Organizational resilience	No special theme is considered, and themes will be found through analysis

Appendix 2

Participants and Interview Overview

**Interview**	**Stakeholder**	**Position**	**Interview method**	**Interview time**
1	AI expert	The founder of a company in developing tools for the healthcare sector and the head in AI tools implementation.	online	01.10.2024<br>29m 15s

2	AI expert	specialized in conversational chatbot solutions based on natural language processing and large language models, designed for the social and healthcare sector	Email	_
3	AI expert	Leader of the AI application development and maintenance team	Email	_
4	AI expert	head of the unit for the AI solutions and data lake services.	Online	10.10.2024<br>42m 8s
5	AI expert	Professor at university with a background mainly in the development of AI models to do image analysis and medical imaging.	online	25.10.2024<br>23m
6	HCP	Specialist and professor in radiology with Professional experience in AI implementation.	Email	_

7	HCP	specializing in general practice and has had cooperation to build medical application	online	23.10.2024<br>34m 53s
8	HCP	Medical physicist and professor at university in medical physics. His team works with AI applications and he collaborates with doctors who use or hope to use such tools.	Email	–
9	HCP	Clinical experience using different AI-enhanced radiograph and MRI image analysis software. Giving Lectures in a related field at university.	Email	–

Appendix 3

Interview Questions for AI Experts

Introduction to the Research:

Hello, and thank you for agreeing to participate in this interview. My name is Zohreh, and I am conducting a study on Key Success Factors in AI Implementation in Healthcare from the Perspectives of Healthcare Professionals and AI experts. This research aims to explore how various factors affect both groups and are influenced by them during the implementation process of AI tools in healthcare and find the gaps between the two groups to improve the cooperation between them.

Consent and Confidentiality:

- The interview will take approximately one hour.
- Your participation in this interview is entirely voluntary, and you may choose to withdraw at any time.
- The interview will be recorded for the analysis in this research.

- The data collected will be used anonymously and will only be stored for the duration of this research project. After the completion of the study, all interview data will be deleted.
- If you consent to participate in this interview, please let me know, and we can proceed.

Structure of the Interview:

Throughout the interview, I will ask questions related to your experience with the implementation of AI tools in healthcare. Please answer them by focusing on the relationship between healthcare professionals and AI experts in your responses, highlighting any gaps or alignments between the two groups for each question. Your input will help us to understand how both groups can better collaborate in this process. Thank you again for your time and valuable insights. Shall we begin?

Questions:

Question 1: would you please explain shortly about your professional experience in AI tools for healthcare?

Question 2: as you know any technology needs adaptation over time to match the organization it is applied to. How important do you think is the cooperation between the healthcare professionals and AI experts in adaptation over time? To what extent do you think healthcare professionals cooperate in this regard?

Question 3: When designing AI tools for healthcare, how important is it for you to ensure that the tool is easy to use for healthcare professionals (considering effort expectancy), or you mostly focus on the functionality of the tool? Which of them you think is more important for healthcare professionals.

Question 4: What do you think about the need for the healthcare professionals to understand the tool and the scope of the use and how you ensure this during implementation process? do you think if healthcare professionals care about it?

Question 5: Do you think if healthcare professionals expect to know how the AI reached a decision or conclusion about a condition (why the algorithm reached a special result and what have been the influential factors) and how you ensure this during development and implementation process?

Question 6: what do you think about the extent to which the healthcare professionals trust AI experts to have the required data and do you think if they cooperate with you in this regard?

Question 7: When developing AI tools for healthcare, how do you ensure the right balance between the tool's independence in decision making and the control that healthcare professionals should maintain over the final decision? What do you think healthcare professionals expect?

Question 8: What do you think healthcare professionals mostly expect the AI tools to do for them, such as reducing repetitive work, improved patient outcomes and consistency or anything based on your experience? To what extent do you care about their expectations?

Question 9: When developing AI tools how do you ensure that the tool helps redefine staff roles in a way that supports rather than disrupts their work and what do you think about healthcare professionals’ expectations in this regard?

Question 10: When developing AI tools, do you care about the extent of change needed for the organization to implement the tool and try to integrate them to the

current practices and workflow along? What are healthcare professionals' expectations?

Question 11: to what extent do you think there is a lack of understanding between healthcare professionals and AI experts in the AI implementation process and if you think there is a need for interdisciplinary experts? Please mention some problems in this regard.

Question 12: How do you address concerns about who is responsible for the outcomes of AI-driven decisions in clinical settings? What do you think about healthcare professionals' expectations in this regard?

Question 13: If you have had experience with the implementation of the AI tools which directly relates to patient care, please also answer the following two questions

13-1 When developing AI for healthcare, how do you ensure that the tool can adapt to different care needs and handle complex or rare decisions?

13-2 How do you ensure that the AI tool can manage patients with multiple health problems (comorbidities)?

Question 14: which of the factors mentioned above is the most important factor for you?

Question 15: is there any other factor that I may have missed?

Appendix 4

Interview Questions for Healthcare Professionals

Introduction to the Research:

Hello, and thank you for agreeing to participate in this interview. My name is Zohreh, and I am conducting a study on Key Success Factors in AI Implementation in Healthcare from the Perspective of Healthcare Professionals and AI experts. This research aims to explore how various factors affect both groups and are influenced by them during the implementation process of AI tools in healthcare and find the gaps between the two groups to improve the cooperation between them.

Consent and Confidentiality:

- The interview will take approximately one hour.
- Your participation in this interview is entirely voluntary, and you may choose to withdraw at any time.
- The interview will be recorded for the analysis in this research.
- The data collected will be used anonymously and will only be stored for the duration of this research project. After the completion of the study, all interview data will be deleted.
- If you consent to participate in this interview, please let me know, and we can proceed.

Structure of the Interview:

Throughout the interview, I will ask questions related to your experience with the implementation of AI tools in healthcare. Please answer them by focusing on the

relationship between healthcare professionals and AI experts in your responses, highlighting any gaps or alignments between the two groups for each question. Your input will help us to understand how both groups can better collaborate in this process. Thank you again for your time and valuable insights. Shall we begin?

Questions:

Question 1: would you please explain shortly about your medical specialty and professional experience with using or implementing AI in healthcare?

Question 2: As you know any technology needs adaptation over time to match the organization it is applied to. How important do you think is the cooperation between the healthcare professionals and AI experts in adaptation of the tool over time? To what extent do you think AI experts care about the adaptation and this cooperation?

Question 3: In your experience using AI tools, how important is the ease of use (effort expectancy) of the AI tool over the functionality? Which one do you think AI experts care about more?

Question 4: In your experience with AI tools, what do you think about the need for healthcare professionals to have a clear understanding of how the tool works and its scope of use? Do you think it is well addressed during the implementation process?

Question 5: What do you think about the need for healthcare professionals to know how AI reached a special decision or conclusion about a condition (which factors have been influential in its decision making) and do you think if it is well addressed by AI experts?

Question 6: What do you think about AI experts accessing high-quality data for AI development and to what extent you dare and trust to collaborate with them in this matter?

Question 7: In your experience with AI tools, what do you think about healthcare professionals’ control over the tool’s decision-making process, and do you think if AI experts care about this matter?

Question 8: What are the performance expectancies of AI tools from the perspective of healthcare professionals (such as reducing repetitive works,

improved patient outcomes and consistency or anything based on your experience) and do you think if AI experts care about your expectations properly?

Question 9: In your experience with AI tools how do you feel about the AI tool's ability to reshape your role and to what extent do you think the AI experts consider them?

Question 10: What do you think about the extent of change needed to your current routines and workflow in case of AI implementation? What do you expect from AI experts in this regard?

Question 11: To what extent do you think there is a lack of understanding between healthcare professionals and AI experts in the AI implementation process and if you think there is a need for interdisciplinary experts?

Question 12: Do you feel clear on who is responsible if something goes wrong while using AI tools? Do you think AI experts care about and address this issue?

Question 13: If you have had experience with the use and implementation of the AI tools which directly relates to patient care, please also answer the following two questions:

13-1 How effectively does the AI tool you use, adapt to different care needs and handle complex or rare decisions?

13-2 How well does the AI tool support you in managing patients with multiple health conditions and what do you expect from AI experts in this regard?

Question 14: which of the factors mentioned above is the most important factor for you?

Question 15: is there any other factor that I may have missed?
